# Reconstructing 3D human pose and shape from a single image and sparse IMUs

**DOI:** 10.7717/peerj-cs.1401

**Published:** 2023-05-24

**Authors:** Xianhua Liao, Jiayan Zhuang, Ze Liu, Jiayan Dong, Kangkang Song, Jiangjian Xiao

**Affiliations:** 1 School of Information Science and Engineering, Ningbo University, Ningbo, China; 2 Ningbo Institute of Materials Technology and Engineering, Chinese Academy of Sciences, Ningbo, China; 3 School of Mechanical Engineering, Zhejiang University of Technology, Hangzhou, China

**Keywords:** 3D human pose and shape, A single image with sparse inertial measurement units, Dual-stream feature extract network, Model-attention network with a residual module, Regression

## Abstract

Model-based 3D pose estimation has been widely used in many 3D human motion analysis applications, in which vision-based and inertial-based are two distinct lines. Multi-view images in a vision-based markerless capture system provide essential data for motion analysis, but erroneous estimates still occur due to ambiguities, occlusion, or noise in images. Besides, the multi-view setting is hard for the application in the wild. Although inertial measurement units (IMUs) can obtain accurate direction without occlusion, they are usually susceptible to magnetic field interference and drifts. Hybrid motion capture has drawn the attention of researchers in recent years. Existing 3D pose estimation methods jointly optimize the parameters of the 3D pose by minimizing the discrepancy between the image and IMU data. However, these hybrid methods still suffer from the issues such as complex peripheral devices, sensitivity to initialization, and slow convergence.

**Methods:**

This article presents an approach to improve 3D human pose estimation by fusing a single image with sparse inertial measurement units (IMUs). Based on a dual-stream feature extract network, we design a model-attention network with a residual module to closely couple the dual-modal feature from a static image and sparse inertial measurement units. The final 3D pose and shape parameters are directly obtained by a regression strategy.

**Results:**

Extensive experiments are conducted on two benchmark datasets for 3D human pose estimation. Compared to state-of-the-art methods, the per vertex error (PVE) of human mesh reduces by 9.4 mm on Total Capture dataset and the mean per joint position error (MPJPE) reduces by 7.8 mm on the Human3.6M dataset. The quantitative comparison demonstrates that the proposed method could effectively fuse sparse IMU data and images and improve pose accuracy.

## Introduction

Reconstructing 3D human pose is important for the somatosensory interaction of gaming, sports and VR/AR applications. With the development of parametric human body models (Anguelov et al., 2005; Loper et al., 2015; Pavlakos et al., 2019), model-based 3D pose estimation methods (Bogo et al., 2016; Kanazawa et al., 2018; Kolotouros et al., 2019; Kundu et al., 2020a; Kundu et al., 2020b; Kocabas et al., 2021) have been developed to obtain the 3D human poses and shapes from images with the prior of some parameterized human model. Most model-based approaches are divided into two broad categories: optimization-based pose estimation methods (Bogo et al., 2016; Pavlakos et al., 2019; Guzov et al., 2021) and learning-based pose estimation methods (Kanazawa et al., 2018; Pavlakos et al., 2018; Kocabas et al., 2021). The optimization-based approach, such as SMPLify (Pavlakos et al., 2019), estimates the model parameters with an iterative optimization process. However, the optimization problem is initial-sensitive and generally slower to converge to an optimum (Ji et al., 2020). The learning-based approach, such as human mesh recovery (HMR) (Kanazawa et al., 2018), uses a neural network to directly regress model parameters from global image features. Nevertheless, it is difficult for the network to learn an effective mapping function, as the parameter space is nonlinear. Benefiting from a pre-defined human model, a complete human pose could be obtained. However, state-of-the-art image-based methods of reconstructing 3D human pose and shape are still sensitive to occlusion. Especially in the outdoor scene, they would produce significantly erroneous predictions, even when most parts of the human body are observable.

Inertial measurement units (IMUs) can effectively solve the viewpoint limitation of accessible optical sensors and produce the rotation information of joint points. Many recent studies (Von Marcard et al., 2017; Yi, Zhou & Xu, 2021; Yi et al., 2022) focus on wearable sensor estimation of body pose by binding some inertial sensing peripherals to key joints of the human body and capturing the direction and acceleration of these joints. However, even if the IMU-based system captures the body movement at a high frame rate, it is susceptible to magnetic field interference and accumulates drift error over time, resulting in exhibit significant position mistakes. Furthermore, the commercial IMU-based system usually relies on dense and complex wearable sensors and time-consuming calibration, *e.g.*, 17 nodes employed by the Xsens Animate suit, causing invasive to the subject and hindering the free movement of the body.

A straightforward manner to improve the estimation accuracy is to combine the individual strengths of image-based and IMU-based methods. Previous works (Li et al., 2017; Majumder & Kehtarnavaz, 2020) have discovered the potential of fusing the features from IMUs and optical cameras for human action recognition. More recently, some robust pose estimation methods (Von Marcard, Pons-Moll & Rosenhahn, 2016; Von Marcard et al., 2018; Malleson, Collomosse & Hilton, 2020; Kaichi et al., 2020) based on IMU and image fusion mainly followed a similar pipeline. These methods generate parametric 3D mannequins and optimize their parameters by minimizing the energy functions associated with IMUs and image features to reduce their differences from images and IMUs. These methods optimize the kinematic pose of the subject based on a cost function comprising orientation, acceleration, 2D position and statistical pose prior terms. A sparse set of IMUs attached to body segments provides the orientation and acceleration constraints. Positional constraints are obtained by 2D joint detections from video cameras. However, this pipeline is initial-sensitive. When 2D joint detection has a small mistake, the accuracy of the predicted pose will significantly decline. To ensure the accuracy of 2D detection, the input sources of these methods are multi-view. The computation time increases exponentially with more input views, as the current 2D joint detector also takes a long time. The exacting requirement also prevents them from being used outdoors or in indoor scenarios with separate rooms.

**Figure 1:**
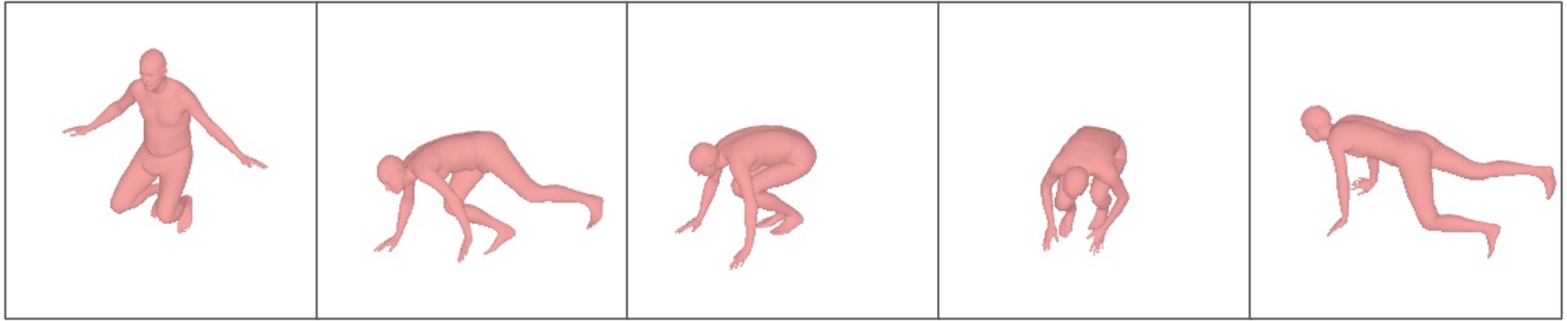
Sample results of reconstructed 3D human pose on the Total Capture dataset. The 3D human pose and mesh estimated from the single-frame image and the sparse IMUs which are presented under camera view.

**Figure 2:**
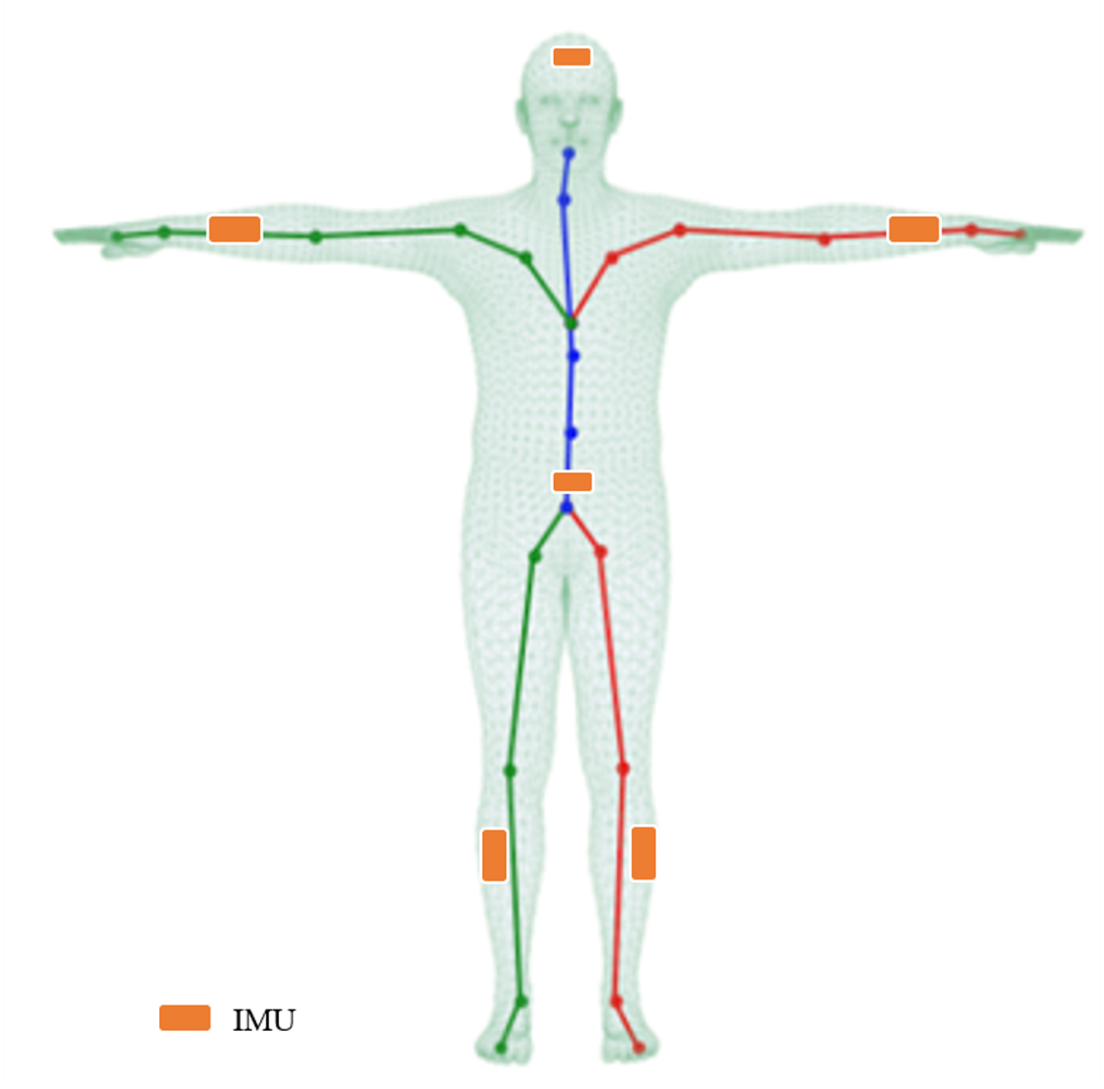
Illustration of the mesh, skeleton, and joints with the T-pose template. The line plots are a common kinematic representation of the human body by 24 keypoints. The triangle mesh is the skinned and vertex-based 3D prior model in SMPL. The placement of six IMUs in this article is indicated by the orange color.

To address the issues mentioned above, this article proposes a method that couples sparse IMUs with a single RGB camera and realizes 3D human body reconstruction through adaptive regression learning. In contrast with SOTA methods based on multi-view and IMUs (Bao, Zhao & Qian, 2022), the proposed approach can avoid the invasion of many IMUs to subjects, avoid 2D joint detection, and reduce hardware dependence. An example of the reconstruction results is shown in Fig. 1. The model in this article realizes robust pose estimation even in the case of severe self-occlusion. An illustration of the IMUs’ binding position is shown in Fig. 2. We keep the same setting of the sparse IMU binding position with previous research (Von Marcard et al., 2017; Huang et al., 2018), and employ a single camera view to provide the auxiliary global information. In other words, this simple setup can obtain stable position information without any drift over time and can calibrate the limb position from the IMUs even suffering from serious occlusion. Besides, a single-view setup like a surveillance camera synergic with several wearable sensors, balancing the limitations of visual occlusion and inertial data drift, is easier to deploy than multi-viewpoint cameras in daily life.

Different from the previous optimization-based method (Von Marcard et al., 2018; Malleson, Collomosse & Hilton, 2020), we adopt a learning-based strategy to fuse the multiple features into a latent vector. A dual-stream network is first used to extract image features and IMU features, respectively. For IMU input, the improved temporal encoder module is applied over a sequence to generate the dynamic features, in which the temporal features are further refined with position embeddings. Simultaneously, a pre-trained single image-based 3D human pose and shape model (Kanazawa et al., 2018; Kolotouros et al., 2019) is employed to extract static image features. The key component of our method is the residual model-attention network, which is used to realize dual-modal feature fusion with the guided-attention from the temporal IMU features and visual features, by introducing a residual branch to connect static image features and fusion features in this article. Finally, the parameterized human pose and body shape are generated by regressing directly from the fused vector.

To evaluate the effectiveness of the proposed approach, we conduct experiments on Total Capture (Trumble et al., 2017; Trumble et al., 2018a; Trumble et al., 2018b) and Human3.6M (Ionescu, Li & Sminchisescu, 2011; Ionescu et al., 2014) datasets, which are commonly used benchmarks for human pose estimation. Compared with the recent PIP (Yi et al., 2022), a sparse-IMU-based method, the per vertex error of our method is reduced by 9.4 mm on the Total Capture dataset. For the Human3.6M dataset, inspired by previous work (Yi, Zhou & Xu, 2021), we synthesize the virtual IMU data and consider measurement error by adding Gaussian noise to validate the general applicability. Compared with the state-of-the-art Mesh Graphormer (Lin, Wang & Liu, 2021), an image-based method, the 3D joint position error goes down by 7.8 mm. The experimental results demonstrate the effectiveness of visual-inertial information fusion.

The main contributions of this article are summarized as follows:

1. This article proposed a hybrid motion capture method in a learning-based framework that combines a single image with sparse IMUs to generate the 3D human pose and shape, which can alleviate the ambiguity and sensitivity of conventional approaches.
2. A residual model-attention network is proposed to closely fuse the dual-stream outputs including dynamic IMU features and static image features. An improved IMU feature extractor is also presented to enhance the robustness against inertial measurement error.
3. The method achieves state-of-the-art performance on two benchmarks for 3D human pose and shape reconstruction, providing a solution for 3D human motion capture in some unrestricted environments in practice.

## Related Work

### Image-based methods

The existing 3D pose image-based estimation approaches can be divided into model-free and model-based methods. The model-free methods (Chen et al., 2019; Wandt & Rosenhahn, 2019; Pavllo et al., 2019) follow a semi-supervised learning manner, mapping 3D keypoints from an image back to 2D keypoints. However, the lack of ground-truth 3D keypoints is the greatest challenge that prevents the composition model from achieving the expected performance in outdoor scenes. Model-based methods yield reasonable pose estimations based on prior knowledge of kinematic models, such as bone-joint connection information, joint rotation characteristics, and fixed bone length ratio (Zhou et al., 2016; Kundu et al., 2020a; Kundu et al., 2020b; Xu et al., 2020). Compared to producing human posture and kinematic bone models, volumetric models can restore high-quality human meshes and provide affiliated shape data of the human body, in which SMPL (Loper et al., 2015) is commonly used in 3D body pose and shape estimation. Several methods (Bogo et al., 2016; Kanazawa et al., 2018; Jiang et al., 2020) proposed use image feature information to regress to SMPL parameters to reconstruct 3D human meshes directly. Other notable approaches (Kocabas, Athanasiou & Black, 2020; Choi et al., 2021; Wei et al., 2022) introduce video-based SMPL body pose and shape estimation. VIBE (Kocabas, Athanasiou & Black, 2020) adversarial training is performed based on the AMASS (Mahmood et al., 2019) large-scale motion capture dataset to distinguish whether the poses predicted by the attitude regression module are consistent with natural human movement postures.

### IMU-based methods

With the development of micro-electromechanical systems, IMUs that measure acceleration and direction have attracted more attention. Some methods have been proposed using only IMUs rather than a marker-based system to restore 3D human posture, such as the commercial inertial motion capture systems (Schepers, Giuberti & Bellusci, 2018) using 17 wearable IMUs to fully obtain the orientation of all bones of the moving body model. However, deploying many sensors is seriously invasive to the subject and hinders the subject’s free movement. In addition, the calibration of multiple sensors often takes a long time. In the methods proposed by Slyper & Hodgins (2008) and Tautges et al. (2011), data from five accelerometers and poses were retrieved from a pre-established motion database. Sparse inertial poser (SIP), a groundbreaking work proposed by Von Marcard et al. (2017), solves human pose estimation using only six IMUs, which is an iterative optimization method that requires access to the entire motion sequence. Deep inertial poser (DIP; Huang et al., 2018) uses a bidirectional recurrent neural network to directly learn body joint rotation information from IMU measurements to SMPL models and provides a DIP-IMU dataset. Transpose (Yi, Zhou & Xu, 2021) and PIP (Yi et al., 2022) use multistage task completion, estimating joint position information before returning to standard rotation information and connecting IMU data to the next stage of the network as an intermediate result, thereby significantly improving accuracy and reducing the running time. Nevertheless, the third stage of Transpose is an inverse kinematics (IK) solver, which would produce keypoint ambiguity. The IK mathematical process that finds relative rotations to create the desired position of body joints, is still an ill-posed problem. Furthermore, simply using the IMU as input to estimate the joint rotation does not introduce any prior knowledge about the human body.

### Image-IMU-based methods

Several works (Von Marcard et al., 2018; Kaichi et al., 2020) have proposed combining images and IMUs to improve the accuracy of 3D human pose and shape estimation. Some methods (Von Marcard, Pons-Moll & Rosenhahn, 2016; Von Marcard et al., 2018; Malleson et al., 2017; Malleson, Collomosse & Hilton, 2020) estimated the 3D human pose by minimizing the joint energy function of the IMUs and image outputs. Gilbert et al. (2019) and Trumble et al. (2017) proposed a dual-stream network that connects the embedding obtained from the image and IMU to obtain the final pose. Zhang et al. (2020) integrated multi-view images and IMUs. The rotation information is fused with image features at an early stage to improve 2D pose estimation directly. In the stage of 3D pose estimation, IMUs data are used to optimize the result through 3D geometry optimization. Although the two-stage method achieves state-of-the-art performance using images alone, it has serious limitations and only works well under an indoor scene with multi-view cameras. Most of the above methods input multi-view images and multiple IMUs, few studies (Von Marcard, Pons-Moll & Rosenhahn, 2016; Von Marcard et al., 2018; Kaichi et al., 2020) reported on pose estimation based on sparse IMUs and single-view images.

In contrast to previous work (Gilbert et al., 2019; Malleson, Collomosse & Hilton, 2020; Zhang et al., 2020), this proposed method in reconstructing 3D human pose lies in two folds: First, instead of estimating the 3D pose from the images or IMUs separately, this article proposes a learning-based framework by incorporating inputs of sparse IMUs and single view images. Second, rather than geometric transformation or 2D-3D lifting, the adopted fusion strategy is feature-level by designing a residual model-attention network, allowing the end-to-end training to generate 3D human pose parameters.

## Method

For clarity, this article first overviews the parametric 3D human body model (SMPL) and introduces the transformation of relevant coordinate systems and data normalization. Then, the following parts introduce the proposed framework and network in detail.

### SMPL body model

SMPL is a skinned and vertex-based 3D prior model of the human body learned from thousands of 3D body scans. The human skeleton is a hierarchy of 24 joints defined by a kinematic tree, which preserves the parenting of the joints. The SMPL model is parameterized by *θ* ∈ ℝ^72^ and *β* ∈ ℝ^10^, where *θ* represents the rotation of the corresponding 23 joints relative to the parent joint and one root (pelvic) global orientation, and *β* is a human morphological vector composed of 10 scalars. Each scalar indicates that the human body expands or contracts in a specific direction. A shape blended T-pose of the SMPL model is shown in Fig. 2. The body mesh *M* ∈ ℝ^*N*×3^can be obtained from the mapping $M \left( \beta ,\theta \right) $ where *N* = 6890. In addition, keypoints *X* ∈ ℝ^*k*×3^ of the human body can also be acquired by a pre-trained linear regressor *W*, *i.e., X* = *WM*, where *k* represents the number of bone points. Thus, the joint positions can be obtained by linearly mapping the mesh vertices with the linear regressor *W*.

### IMU calibration and normalization

#### Calibration

**Figure 3:**
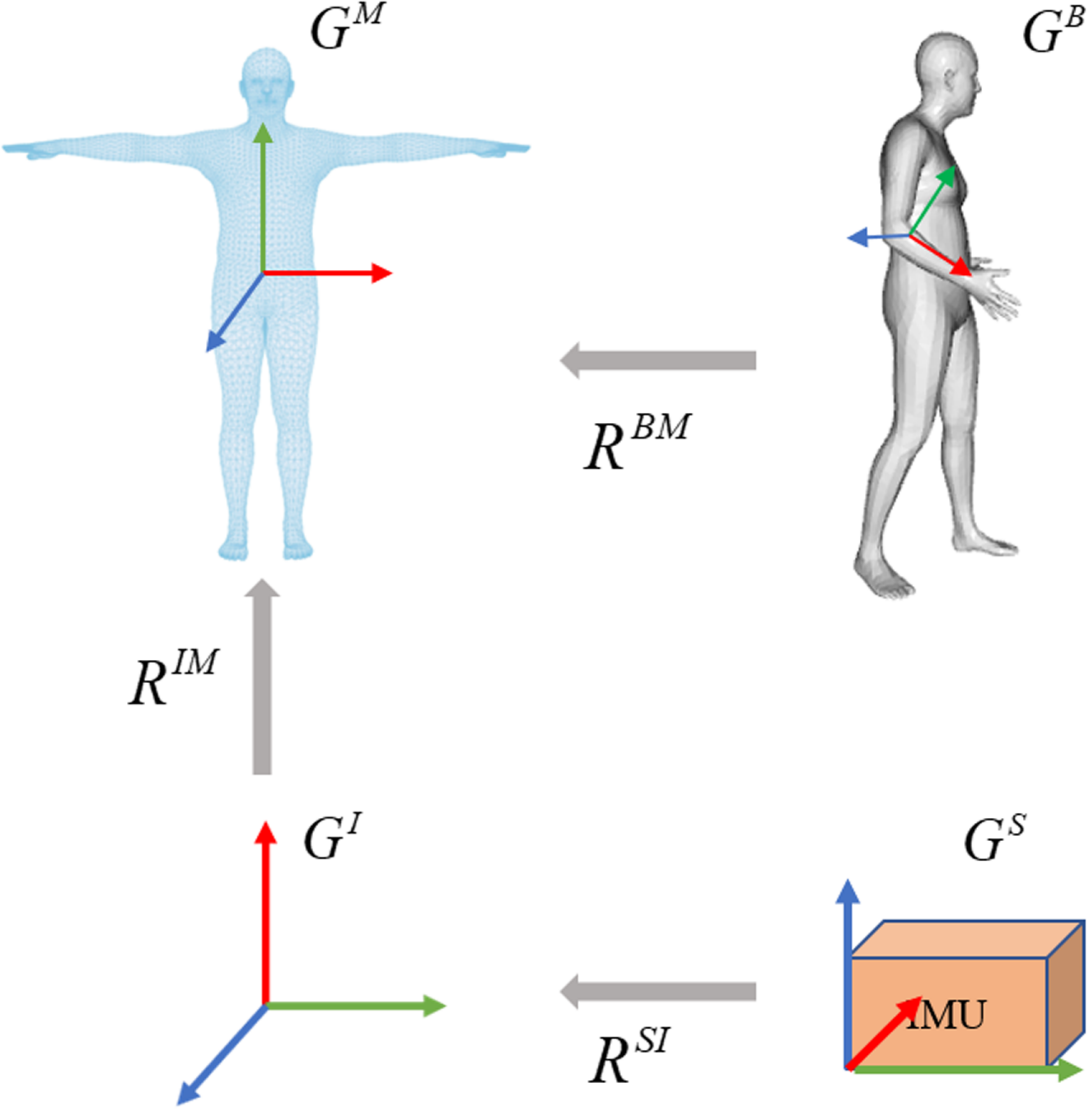
Overview of the calibration process on coordinate frames. The calibration involves three steps. The sensor coordinate system is transformed into the global inertial coordinate system. The global inertial coordinate system is then transformed into the SMPL global inertial coordinate system. Due to the inherent offset between the IMUs and the corresponding joints in SMPL, the third step is to calculate the joint rotations by giving a known template such as the T-pose.

The overview of IMU calibration is illustrated in Fig. 3. Referring to DIP (Huang et al., 2018), the calibration aims to transform the IMU readings to a common body-centric coordinate frame. Denoting the acceleration data relative to the sensor coordinates as *G*^*s*^ and the orientation data relative to the global inertial coordinates as *G*^*I*^. To convert the inertial measurement value to the SMPL global coordinate frame *G*^*M*^, it is necessary to calibrate the coordinate sensor frame to the global inertial coordinate frame, represented as a rotation *R*^*SI*^: *G*^*S*^ → *G*^*I*^. From the global inertial coordinate frame to the 3D human body model (SMPL) global coordinate frame, it is defined as a rotation *R*^*IM*^:*G*^*I*^ → *G*^*M*^. Supposing *G*^*I*^ and *G*^*M*^ are unchanging during the calibration period, the process is expressed as:

(1) \begin{eqnarray*}{G}^{M}={R}^{MI}{G}^{I},\end{eqnarray*}

where *R*^*MI*^ = *inv*(*R*^*IM*^) represents the transformation of the global inertial coordinate to the SMPL global coordinate.

The bone offset under the SMPL global coordinate is necessary as the sensor can be placed in any direction. At the beginning of each sequence, each subject stands in a known T-pose with bone orientation ${O}_{0}^{BI}$ and acceleration ${A}_{0}^{BI}$. The orientation offset ${R}_{offset}^{BM}$ and acceleration offset ${A}_{offset}^{BM}$ are assumed to be unchanged during the calibration period, the per-sensor bone offsets are computed by:

(2) \begin{eqnarray*}{R}_{offset}^{BM}=inv({O}_{0}^{IB}){R}^{IM},\end{eqnarray*}

(3) \begin{eqnarray*}{A}_{offset}^{BM}={A}_{0}^{BI}{R}^{IM}.\end{eqnarray*}

Based on bone offset, the orientation and acceleration data of each sensor is first transformed to the global inertial coordinates, then converted to the SMPL global coordinates. This process is expressed as:

(4) \begin{eqnarray*}{O}_{t}^{BM}={O}_{t}^{BI}{R}^{IM}{R}_{offset}^{BM},\end{eqnarray*}

(5) \begin{eqnarray*}{A}_{t}^{BM}=({A}_{t}^{BI}{R}^{IM})-{A}_{offset}^{BM},\end{eqnarray*}

where *O* ∈ ℝ^3×3^ represents the orientation and *A* ∈ ℝ^3^ indicates the acceleration, ${O}_{t}^{BM}$ and ${A}_{t}^{BM}$ represent the direction and acceleration of *t*-th frame in the SMPL global coordinates, respectively. ${O}_{t}^{BI}$ and ${A}_{t}^{BI}$ represent the direction and acceleration of *t*-th frame in the global inertial coordinates, respectively. Then the normalized ${O}_{t}^{BM}$ and ${A}_{t}^{BM}$ are used as input for the improved IMU feature extractor.

#### Data normalization

After converting the IMU readings to the SMPL coordinate frame, denote the normalized direction and acceleration as $\overline{O}$ and $\overline{A}$, respectively. The leaf joint inertial measurements are aligned with the root joint:

(6) \begin{eqnarray*}{A}_{leaf}={\overline{O}}_{leaf}^{-1}({\overline{A}}_{leaf}-{\overline{A}}_{root}),\end{eqnarray*}

(7) \begin{eqnarray*}{O}_{leaf}={\overline{O}}_{root}^{-1}{\overline{O}}_{leaf}.\end{eqnarray*}

The standard root is defined as:

(8) \begin{eqnarray*}{A}_{root}={\overline{O}}_{root}^{-1}{\overline{A}}_{root},\end{eqnarray*}

(9) \begin{eqnarray*}{O}_{root}={\overline{O}}_{root}.\end{eqnarray*}

### Network architecture

**Figure 4:**
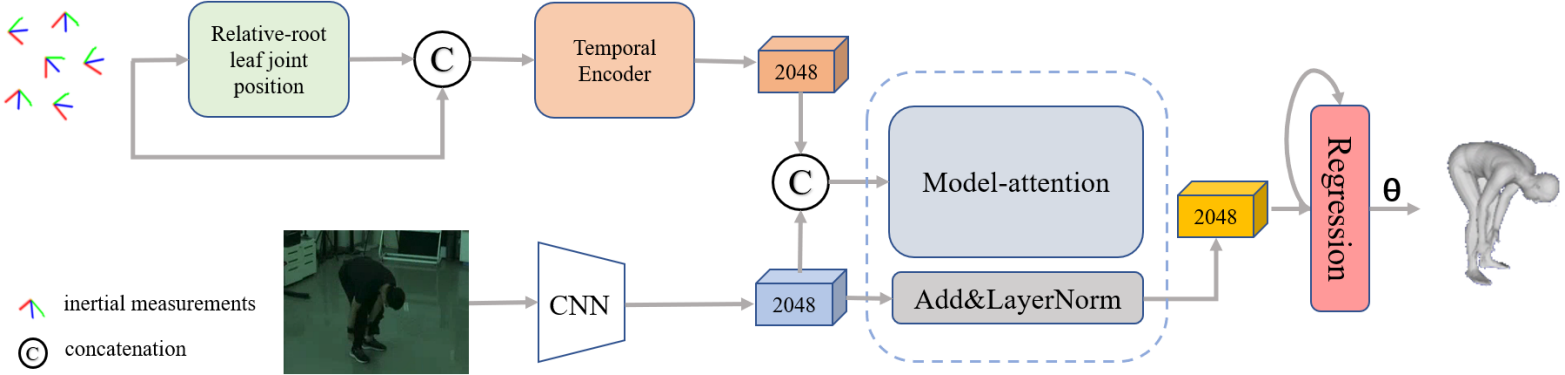
Overview of our proposed framework for reconstructing the human pose and shape from a single image and sparse IMUs. The pipeline contains three stages: a dual-stream network consisting of an improved IMU feature extractor and a general image feature extractor, a residual model attention-based that is implemented to fuse the multiple features and a regression network for 3D human pose generation.

The pipeline of our proposed network is shown in Fig. 4, which mainly consists of three stages. The first stage is a dual-stream network, including a temporal encoder to extract dynamic IMU features and a simple convolutional neural network with pre-trained weights as the image encoder to extract the visual features. The second stage is a model attention network for fusing features from different modalities, and the third stage is a regression network for generating pose parameters from the fused feature vector.

#### Dual-stream feature extractors

**Figure 5:**
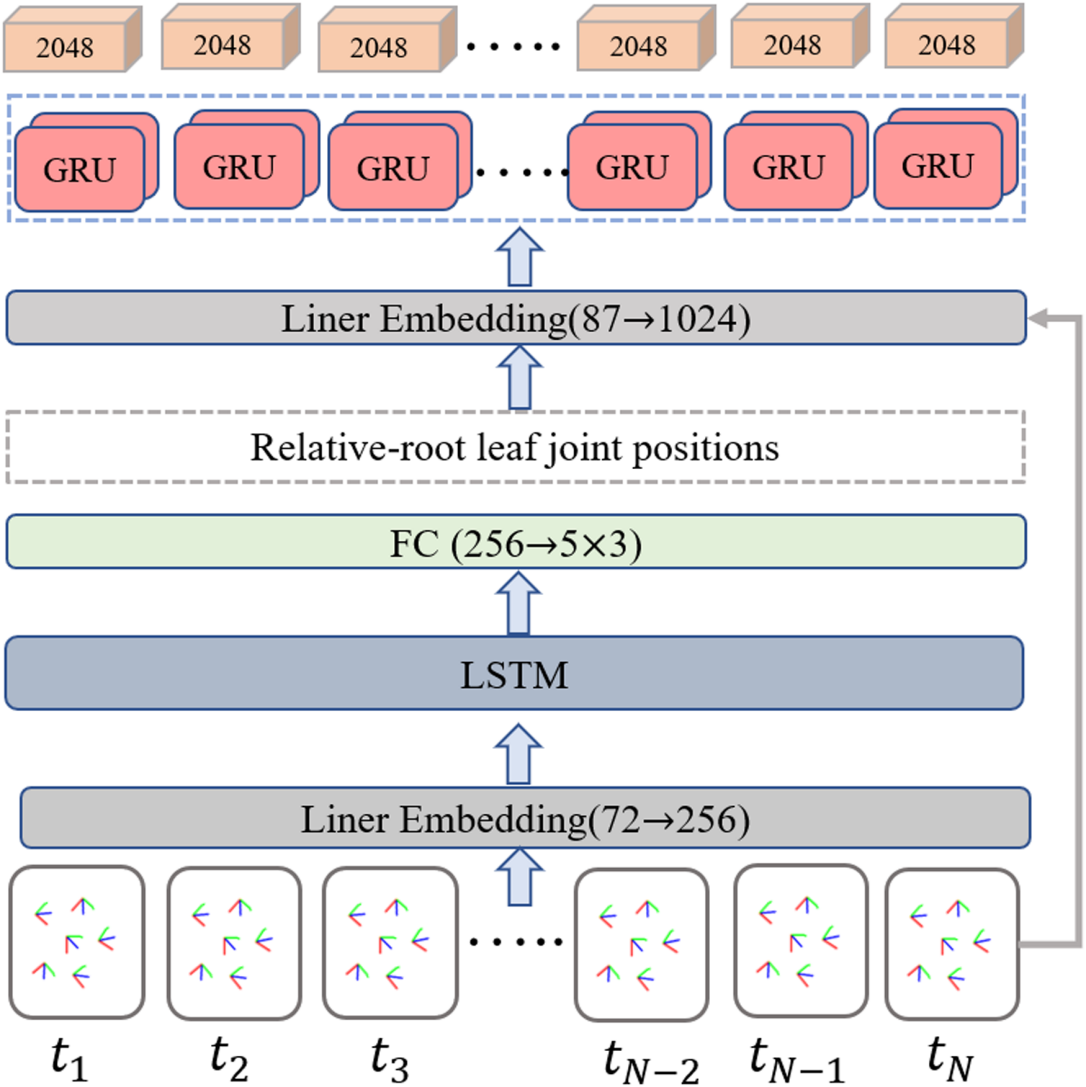
Overview of the improved IMU feature extractor. The relative root leaf joint positions are determined as an intermediate output through the implementation of a regular bi-directional LSTM-based network. The additional temporal encoder equipped with a two-layer bidirectional gated recurrent unit (GRU) is incorporated to further refine the temporal feature using position embeddings and generates T×2048 features as the final output.

To balance the efficiency, scalability, and long-term modeling ability to extract representative information from the temporal inertial data, the bidirectional recurrent neural network (Schuster & Paliwal, 1997) with long short-term memory (LSTM) (Hochreiter & Schmidhuber, 1997) unit is employed as the IMU feature extractor. A detailed implementation of the IMU feature extractor is illustrated in Fig. 5. The input data is a temporal sequence containing the normalized positions of the leaf joint relative to the root. The output of the IMU feature extractor is a set of the corresponding frame-level feature vector. Specifically, a linear embedding layer is first used to encode the normalized position data, then a two-layers LSTM is employed to aggregate the temporal cues in the context. The intermedia feature of five leaf joint positions relative to the root joint is obtained through a fully connected layer. Inspired by the position encoding scheme proposed by Zhao, Wang & Tian (2022) in image-based keypoint detection, we add an extra temporal encoder containing a two-layer bidirectional gated recurrent unit (GRU) to refine the temporal feature with position embeddings and generate T ×2048 features as output.

**Figure 6:**
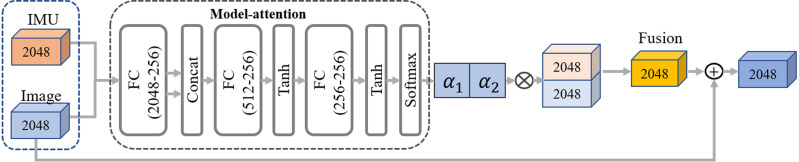
The pipeline of our residual model attention-based network. The network takes in two frames of IMU and image input features, following the primary pathway to generate the fusion features. Additionally, two residual connections including one from the static image features and the other from the temporal IMU feature, are concatenated to the fused feature in turn, further enhancing feature representation capability. Two residual connections are also concatenated to the fused feature in turn.

For the image-based input branch, we refer to the previous single image-based method (Kanazawa et al., 2018; Kolotouros et al., 2019) and employ the ResNet-50 (He et al., 2016a; He et al., 2016b) based backbone with pre-trained weights by the ImageNet as an image encoder to extract the visual feature *f*_*img*_ ∈ ℝ^2048^. Then the extracted visual feature is processed by two branches. As the cubes in blue color shown in Fig. 6, the first one is used as the input to fuse with the IMU features in the residual model-attention network, while the second one is connected to the final regression module to obtain the pose parameter *θ* and body parameter *β*.

#### Residual model-attention network

To closely couple distinct dual-modal features and eliminate ambiguity, we design a residual model-attention network to refine the image feature guided by the IMU feature. As shown in Fig. 6, the fusion network combines a model-attention network and a residual connection network. The input of model attention is a distinct dual-modal feature, and the output is a fused feature aggregated from visual image and IMUs. Referring to the residual connection introduced by He et al. (2016a) and He et al. (2016b), the fused feature $f \left( t \right) $ is generated by concatenating the model-attention feature ${f}_{attention} \left( t \right) $ with the original IMU feature ${f}_{imu} \left( t \right) $ and image feature ${f}_{img} \left( t \right) $ in turn. The detailed process is expressed as:

(10) \begin{eqnarray*}{f}_{attention}^{{^{\prime}}} \left( t \right) =Max \left( {f}_{attention} \left( t \right) ,{f}_{imu} \left( t \right) \right) ,\end{eqnarray*}

(11) \begin{eqnarray*}f \left( t \right) =LayerNorm \left( {f}_{attention}^{{^{\prime}}} \left( t \right) +{f}_{img} \left( t \right) \right) ,\end{eqnarray*}

where $Max \left( \cdot \right) $ indicates the max pooling operation, $LayerNorm \left( \cdot \right) $ indicates the layer normalization inspired by previous work (Yu et al., 2019).

The final regression module takes in the fused feature $f \left( t \right) $ and generates a set containing pose parameter *θ* and body parameter *β*. Following the iterative regression module introduced in Kolotouros et al. (2019), the regression network is initialized with an average posture, then the output pose *θ* is regressed with the input fused feature $f \left( t \right) $ through an iterative process.

#### Loss function definition

As SMPL is a differentiable digital model that can generate 3D keypoints by a linear regression $\hat {X} \left( \theta \right) =WM \left( \theta ,\beta \right) $, where $\hat {X} \left( \theta \right) \in {\mathbb{R}}^{J\times 3}$ and *J* represents the number of joints. The body mesh $\hat {V} \left( \theta \right) =M \left( \theta ,\beta \right) $ can be also generated by linear blend skining, where $\hat {V}\in {\mathbb{R}}^{6890\times 3}$, indicating that the mesh consists of 6,890 points. Therefore, the constraints of the endpoint regression module include the mesh, 3D keypoints, pose parameters *θ*, and shape parameters *β*. The loss function for training the end regression network is defined as:

(12) \begin{eqnarray*}{L}_{c}=\tau {L}_{vertices}+\varphi {L}_{keypo\mathrm{int}s}+w{L}_{SMPL},\end{eqnarray*}

where *L*_*vertices*_ denotes the body mesh loss, *L*_*keypoints*_ denotes the 3D keypoints loss, and *L*_*SMPL*_ denotes the pose parameter loss. *τ*, *φ* and *w* denote the corresponding weights of three loss items. These losses are denoted as follows:

(13) \begin{eqnarray*}{L}_{vertices}={|}{|}V-\hat {V}{|}{{|}}_{1},\end{eqnarray*}

(14) \begin{eqnarray*}{L}_{keypo\mathrm{int}s}={|}{|}X-\hat {X}{|}{{|}}_{2},\end{eqnarray*}

(15) \begin{eqnarray*}{L}_{SMPL}={|}{|}\beta -\hat {\beta }{|}{{|}}_{2}+{|}{|}\theta -\hat {\theta }{|}{{|}}_{2},\end{eqnarray*}

where *θ* ∈ ℝ^24×3×3^, and *V*, *X*, *β* and *θ* represent ground truths of $\hat {V},\hat {X},\hat {\beta }$ and $\hat {\theta }$, respectively.

To better utilize the relative joint position information calculated by IMU, we add an intermedia constraint for the IMU feature extractor during the end-to-end training. Denote the leaf joints containing five relative-root joints in a sequence as ${J}_{leaf}= \left[ {J}_{r\text{_}ankle},\ldots ,{J}_{l\text{_}wrist} \right] $. The loss item for the IMU feature extractor is defined as:

(16) \begin{eqnarray*}{L}_{imu}=\sum _{t}^{T}{|}{|}{J}_{leaf}-{J}_{leaf}^{GT}{|}{|}.\end{eqnarray*}

The overall objective is defined as:

(17) \begin{eqnarray*}L={L}_{c}+\lambda {L}_{imu},\end{eqnarray*}

where *λ* is the corresponding weight to control the relative importance.

### Implementation details

Following previous work, the human bounding box is used in both the training and testing phases for image-based input. The cropped image containing the human body was resized to 224 × 224.

Following the IMU measurement processing of Transpose (Yi, Zhou & Xu, 2021), the acceleration was scaled to 30 times of the original value, normalized and flattened into 72-dimensional vector. The sequence length of IMU data is *T* = 16 to aggregate the temporal cues in the context. For network training, the learning rate was initially set to 5 × 10^−5^ with using the Adam optimizer, training the network for a total of 50 epochs on two GPUs. The batch size is set to 32, and the weights of the loss item are *τ* = 1 × 10^−2^, *φ* = 1, *w* = 2 × 10^−1^, *λ* = 1 × 10^−2^, respectively. PyTorch was used for code implementation.

## Experiments

### Datasets and metrics

#### Data preparation

The Total Capture dataset is currently the only dataset that provides images, IMU data, and ground-truth annotations for 3D keypoints. Its data contain images captured by eight cameras and 13 IMUs (containing rotation information and acceleration). This dataset contains four actions performed by five subjects, with each action repeated three times. The experiment used images taken by the eighth camera and values from six IMUs as input to the proposed network. Experiments on this dataset follow the protocol proposed by previous work (Zhang et al., 2020), the movements roaming 1, 2, and 3; walking 1 and 3; acting 1 and 2; and freestyle 1 and 2 of the first three subjects is used as the training set. The actions of the five subjects (walking 2, acting 3, and freestyle 3) are used for evaluation. Following the IMU sensor setting of DIP (Huang et al., 2018), the selected six IMUs’ rotation and acceleration are provided by the authors of DIP.

As the Human3.6M dataset does not provide IMU data, we follow the evaluation setup of DIP (Huang et al., 2018) to validate the general applicability of this fusion strategy. The synthetic IMU training data, *i.e.,* the accelerations and orientations of six keypoints indicated by the orange color in Fig. 2, is obtained by creating virtual sensors on the SMPL mesh surface. The global rotation is generated by local rotation through forward kinematics (FK), where the global rotation is the orientation of the IMU. The acceleration of the virtual sensor is computed through finite differences. More precisely, assuming the position of a virtual IMU is *p*_*t*_ for time *t,* and the time interval between the two frames is *d*_*t*_, the fitted acceleration can be calculated by

(18) \begin{eqnarray*}{a}_{t}= \frac{{p}_{t-1}+{p}_{t+1}-2\times {p}_{t}}{{d}_{t}^{2}} ,\end{eqnarray*}

(19) \begin{eqnarray*}{R}_{IMU}=FK(\theta ,\beta ).\end{eqnarray*}

The Human3.6M dataset contains seven subjects with 15 sets of actions, with each set repeated twice. The SMPL parameters were taken from the same source as the previous HybrIK (Li et al., 2021) and L2LMeshNet (Moon & Lee, 2020). Following HMR and SPIN (Kanazawa et al., 2018; Kolotouros et al., 2019), this article used the first five subjects for training and the last two subjects for evaluation.

#### Evaluation metrics

For a fair comparison, we employ the most common metrics to report the experimental results. The evaluation metrics are defined as follows:

1. SIP error measures the global rotation error of the upper arm and thigh in degrees.
2. The angle error indicates the global rotation error of each body joint in degrees.
3. MPJPE represents the average distance error of the node in mm, aligned with the root joint.
4. PA-MPJPE indicates all nodes’ average Euclidean distance error after further rigid alignment in mm.
5. PVE means mesh error; the unit is cm, representing the average error of the vertex in the mesh model. It is a better representation of deformation error.

### Ablation study

To evaluate the effectiveness and robustness of this method to improve 3D pose and shape estimation, the ablation study discusses the impact of multimodal feature fusion, influence of the IMUs’ placement on individual joints, and analysis on IMU drift with simulated noise in detail. Note that IMU data here are calibrated and normalized directions and accelerations defined by Eqs. (8) and (9), respectively.

#### Analysis of feature fusion

This article aims to investigate the efficacy of fusing IMU and image in improving the accuracy of 3D pose estimation. To this end, the Total Capture dataset containing ground-truth annotations is employed as the benchmark. This ablation study was conducted by giving four different inputs, designated as “IMU only”, “Image only”, “IMU+Image (TP)”, and “IMU+Image” employing four evaluation metrics including reconstructed joint, body mesh, and angle errors. The detailed experimental settings with four different inputs are as follows:

1. “IMU only” follows Transpose as the baseline, which uses the LSTM network to obtain leaf keypoints and all relative-root keypoints, connect IMU measurements, and generate IMU features. The regressor is then used to generate pose parameters.

2. “Image only” follows HMR as the baseline, which uses the single-view image feature as input to the regression network to generate pose parameters.

3. For the “IMU+Image (TP)”, involved IMU feature and image feature follow the above “IMU only” and “Image only” baselines. “TP” represents the IMU feature extractor following TransPose (Yi, Zhou & Xu, 2021). The fusion scheme defined in Eqs. (10) and (11) is employed to generate pose parameters.

4. In contrast to “IMU+Image (TP)”, “IMU+Image” used the LSTM network to obtain the leaf keypoints of the relative root as the intermediate result, connecting the IMU measurement value and generating a combined IMU feature. The fusion scheme follows ”IMU+Image(TP)”.

The detailed results are listed in Table 1. Overall, the experimental results demonstrate that the fusion of IMU and image features, referred to as “IMU+Image (TP)” and “IMU+Image”, significantly improves pose estimation accuracy. The superiority on all metrics indicates that the proposed network can effectively couple the different features and reduce the 3D pose and shape error. In addition, the results of “IMU+Image” are further better than those of the “IMU+Image (TP)”, which that indicates the improved IMU feature extractor is more robust to the sensor disturbance on aggregating the temporal information. “IMU+Image (TP)” depends on a leaf joint’s target position, and the position information is reverse derived for *n* parent joints on the bone chain where it is located to determine the position of the entire bone chain. However, this derivation process is sensitive to the incorrect feature, resulting in an inaccurate pose position.

#### Influence of the IMUs on individual joints

Specifically, to explore the effects of IMUs on improving the accuracy of each joint position, the method was evaluated by reconstructing 14 key joints defined by Leeds Sports Pose (LSP, Johnson & Everingham, 2010) with taking in IMU data as input or not. Following the previous work (Kanazawa et al., 2018; Kolotouros et al., 2019), the 14 LSP joints were regressed from the body mesh by a pre-trained regressor. The main result is shown in Table 2. The auxiliary use of IMUs results in a significant reduction in position errors of six key joints. In particular, the error of wrist and ankle joints reduce by 29.5% and 34.3%, respectively. The end joints, such as the ankle and wrist, are more sensitive to visual occlusion due to the flexible movement of the human body and the limitations of the camera view. This experimental result indicates that the number of IMUs could be further reduced to correct the estimated error of image-based motion capture for specific applications like virtual action sports games.

#### Analysis of IMU drift with noise

As the IMU is sensitive to the magnetic field and noisy during the measurement, resulting in prediction jitter and drift. To address this issue, this section mainly discusses the effectiveness of fusing IMU and image features in overcoming measurement noise. Inspired by TransPose (Yi, Zhou & Xu, 2021), this experiment was conducted on the Human3.6M dataset to simulate the IMU noise quantitatively, as the ideal data could be generated by inferring the positions and rotations of a virtual IMU on the corresponding vertices of the SMPL mesh. Note that we add Gaussian noise to the virtual IMU data during the test phase. Three different standard deviations *i.e.,* 0.12, 0.2, 0.3 were randomly added to the 30% of the raw IMU data to simulate the noisy measurements in real-world settings. The experimental results are presented in Table 3. The ablation study compares two types of input data, which are denoted as “IMU only” with four standard deviations and “IMU+Image” with four standard deviations, respectively.

**Table 1:** Evaluation of the different input data for pose estimation on the Total Capture dataset. The evaluation metrics include SIP, Angle, MPJPE and PVE indicate the mean global Angle error for the shoulder and hip joints, the mean global Angle error, the mean position error for all joints, and the body mesh error, respectively.

**Input data**	**SIP(deg)**↓	**Angle(deg)**↓	**MPJPE(cm)**↓	**PVE(cm)**↓
IMU only	14.11	15.09	7.27	8.24
Image only	12.08	15.23	5.86	6.82
IMU+Image (TP)	10.52	12.28	5.04	5.80
IMU+Image	10.18	12.02	4.88	5.57

**Table 2:** Evaluation of the position error of each joint with and without IMU data. The samples of subject 1 from the Total Capture dataset are selected as the benchmark with MPJPE in mm reported for six key joints of the human body.

**Joint**	**Hip**	**Knee**	**Ankle**	**Shoulder**	**Elbow**	**Wrist**
Image	9.25	40.22	70.21	37.15	54.10	88.34
IMU	25.50	40.71	75.05	41.07	60.78	75.08
Image+IMU	9.02	29.26	46.10	28.72	43.45	62.25

**Table 3:** Evaluation of the impact of noisy IMU data on predicted pose accuracy with and without Image input. The virtual IMU data calculated from the Human3.6M dataset is utilized as the baseline to assess the effect of noisy data. Gaussian noises were added with varying standard deviations including 0.12, 0.2 and 0.3. The metrics of PA-MPJPE and MPJPE in mm are reported with and without Image input.

Input data	Noise value	PA-MPJPE (mm)	MPJPE (mm)
IMU only	0.12	61.66	84.55
IMU only	0.20	62.51	85.53
IMU only	0.30	67.24	91.75
IMU only (without noise)	–	55.87	75.75
IMU+Image	0.12	37.39	49.64
IMU+Image	0.20	37.63	49.80
IMU+Image	0.30	37.78	50.04
IMU+Image (without noise)	–	33.34	43.44

Compared to noiseless IMU data, the predicted pose error with using noisy IMU data would increase by about 27.7%. However, the pose error predicted by fusing images and noisy IMU data only increased by about 15.1%. By adding the same IMU noise, the joint accuracy of the fused method improves by about 45.4% compared to that of the single-source IMU data. The superiority in improving joint accuracy even with noisy inference demonstrates that the proposed fusion network is qualified for reducing the ambiguity and drift of conventional approaches.

### Comparison with state-of-the-art

To the best of our knowledge, only a few works (Von Marcard, Pons-Moll & Rosenhahn, 2016; Von Marcard et al., 2018; Kaichi et al., 2020) have addressed pose estimation using sparse IMUs and a single camera. To further demonstrate the advantages of this work over single and multiple data-based approaches, the proposed method is compared with previous pose estimation methods from three folds: sparse IMUs, single-frame image, and fusion methods.

#### Comparison with sparse IMUs-based methods

To demonstrate the superiority of our improved IMU feature extractor in encoding the temporal information, the experiment employs the Total Capture dataset as the benchmark and compares the results with the recent methods based on sparse IMUs. The quantitative comparison between state-of-the-art methods and ours on the Total Capture dataset is shown in Table 4. The metrics of SIP, Angle error, MPJPE, and PVE are used to evaluate the predicted human pose and body mesh. For the Angle error, the AAGC-LSTM network proposed by Puchert & Ropinski (2021) performs better. However, the model presented in this article is superior in the other metrics. The results outperform those of the latest method PIP (Yi et al., 2022). Especially, the per-vertex error (PVE) is reduced by 14.4%, indicating the effectiveness of the proposed approach.

**Table 4:** Comparison with the sparse IMUs-based state-of-the-art methods on the Total Capture dataset. The evaluation is conducted by SIP, Angle, MPJPE and PVE metrics. “–” indicates the results are not available from the original article. The results of “Ours (TP)” and “Ours” are based on the settings described in the ablation study.

**Method**	**SIP (deg)**↓	**Ang (deg)**↓	**Pos (cm)**↓	**PVE (cm)**↓
Yi, Zhou & Xu (2021)	17.39	17.87	7.43	8.27
Puchert & Ropinski (2021)	13.12	10.12	6.00	–
Yi et al. (2022)	12.93	–	–	6.51
Ours (TP)	10.52	12.28	5.04	5.80
Ours	10.18	12.02	4.88	5.57

**Figure 7:**
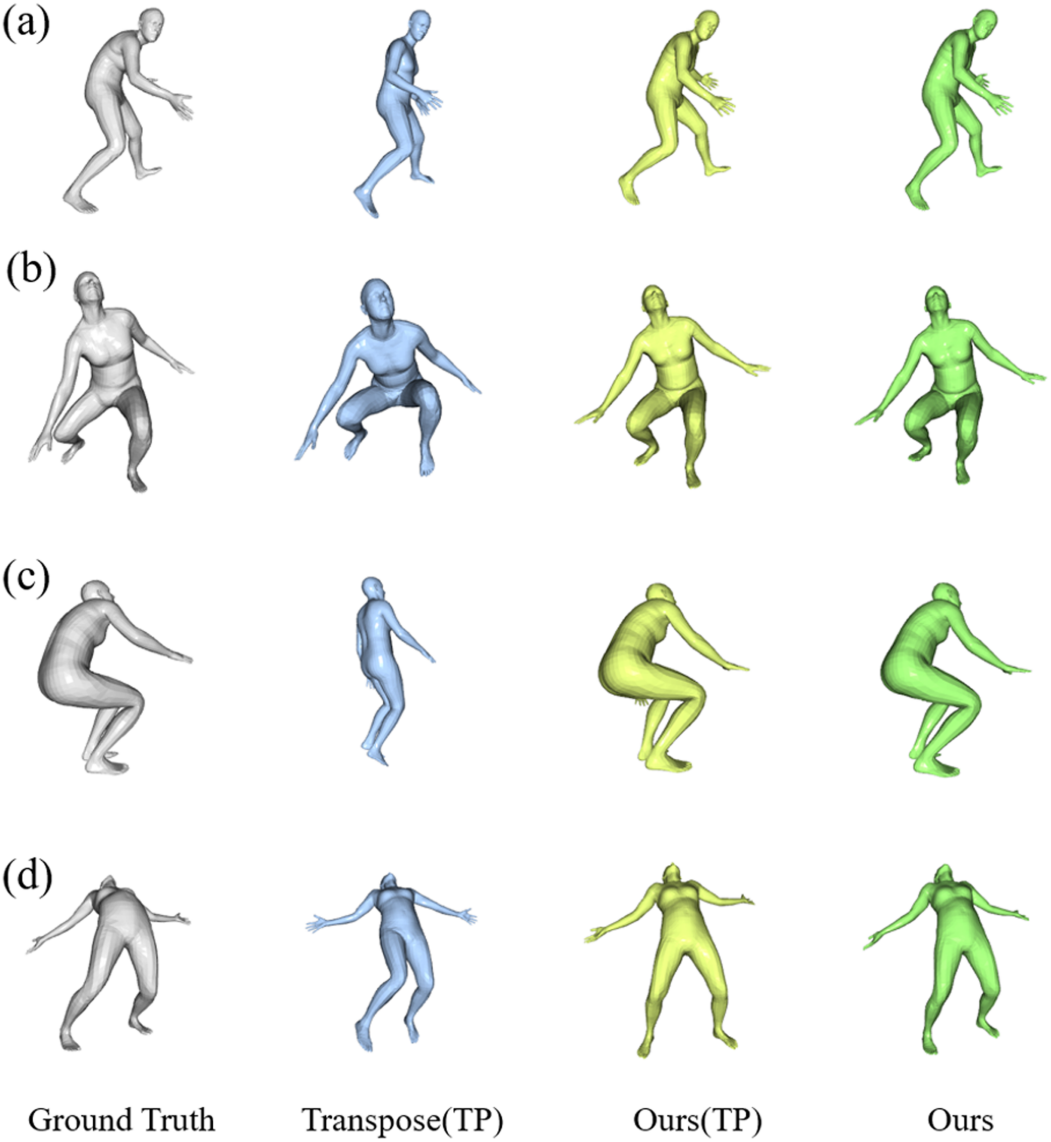
Qualitative results of our results compared to other methods on the Total Capture dataset. The results are “Ground Truth”, “Transpose”, “Our (TP)” following the Transpose, and “Our” from left to right. The samples of (A) and (B) are hand-picked from the results of TransPose with a higher score. Sample (C) is a failure case of TransPose and (D) shows a case chosen from the reconstruction of our proposed model with the lower score.

Figure 7 presents some visualization results of our method and Transpose (Yi, Zhou & Xu, 2021) on the Total Capture dataset. The SMPL model is projected to the same view for better visual effects of human posture. The cases shown in the top two rows are selected from the outputs of Transpose (Yi, Zhou & Xu, 2021) with relatively high scores. The case shown in the third row is a representative ambiguous frame, and the bottom row shows a case chosen from the reconstruction with a lower score by our proposed model. As shown in the first- and second-line examples in Fig. 7, while each model is correct in rough structure, the model is much better at reconstructing arm and leg position details, such as step span, leg bend, and visual arm posture. The model performed better when reconstructing the pose with both legs bent, as shown in the third example of squatting below; the reconstruction is visually closer to the ground truth than the Transpose (Yi, Zhou & Xu, 2021). In the fourth example, each model fails to produce the correct pose, but our model still achieves much better results in the detail of the leg position.

#### Comparison with image-based methods

Following previous work (SPIN and HMR), this experiment evaluated the reconstructed 14 LSP joints on the Human3.6M dataset compared with previous methods based on single-view images. Table 5 lists the detailed results using the rigid alignment average position error per joint (PA-MPJPE) and position error per joint (MPJPE). As shown in Table 5, this method could reduce the joint error (MPJPE) by 13.5% compared to the SOTA method.

To further analyze the influence of partial occlusions on pose estimation, the detailed results on various human postures with performing 15 movements are listed in Table 6. Note that the results of several methods using multi-view images are also given for a fair comparison. This method outperforms better for most actions, particularly the refine actions such as “Eating” and “Photo” which are significantly corrected benefits from the auxiliary virtual IMU information. The accuracy for general actions like “Sitting Down” and “Walking Dog” is inferior to that of single-view methods. The main reason is the proposed method does not consider the global translation, resulting in the estimated position error.

**Table 5:** Comparison with the monocular image-based state-of-the-art methods on the Human3.6M dataset. The comparison is conducted by two metrics including PA-MPJPE and MPJPE in mm. “*” indicates that the method was not pre-trained on the different combinations of datasets, and “–” indicates the results are not available from the original article.

**Methods**	**PA-MPJPE(mm)**↓	**MPJPE(mm)**↓
HMR (Kanazawa et al., 2018)	56.8	88.0
SMPLify (Pavlakos et al., 2019)	82.3	–
SPIN (Kolotouros et al., 2019)	41.1	–
Pose2Mesh (Choi, Moon & Lee, 2020)	47.0	64.9
L2LMeshNet* (Moon & Lee, 2020)	41.7	55.7
Lin, Wang & Liu (2021)	34.5	51.2
HybrIK (Li et al., 2021)	34.5	54.4
Ours	33.3	43.4

**Table 6:** Comparison of the results of 15 movements with other approaches on Human3.6M. The metric is MPJPE in mm. Both multiview-based and single-view with additional training data are considered for the evaluation.

Method	Multiview	Direct	Discus	Eating	Greet	Phone	Photo	Pose	Purch.
Malleson et al. (2017)	Yes	92.7	85.9	72.3	93.2	86.2	101.2	75.1	78.0
Martinez et al. (2017)	Yes	74.0	94.6	62.3	59.1	65.1	49.5	52.4	62.9
Trumble et al. (2018a) and Trumble et al. (2018b)	Yes	61.0	95.0	70.0	62.3	66.2	53.7	52.4	62.5
Gilbert et al. (2019)	Yes	61.2	63.0	58.6	91.2	76.3	91.1	59.7	68.3
Zhao et al. (2019)	No	48.2	60.8	51.8	64.0	64.6	53.6	51.1	67.4
Ci et al. (2019)	No	46.8	52.3	44.7	50.4	52.9	68.9	49.6	46.4
Liu et al. (2020)	No	46.3	52.2	47.3	50.7	55.5	67.1	49.2	46.0
Xu & Takano (2021)	No	45.2	49.9	47.5	50.9	54.9	66.1	48.5	46.3
Zhao, Wang & Tian (2022)	No	45.2	50.8	48.0	50.0	54.9	65.0	48.2	47.1
Ours	No	37.2	42.6	38.5	43.0	43.0	49.7	39.3	39.4
Method	Multiview	Sitting	SittingD.	Smoke	Wait	W.Dog	Walk	W.toget	Mean
(Malleson et al., 2017)	Yes	83.5	94.8	85.8	82.0	114.6	94.9	79.7	87.3
Martinez et al. (2017)	Yes	74.0	94.6	62.3	59.1	65.1	49.5	52.4	62.9
Trumble et al. (2018a) and Trumble et al. (2018b)	Yes	61.0	95.0	70.0	62.3	66.2	53.7	52.4	62.5
Gilbert et al. (2019)	Yes	76.2	93.4	71.2	85.0	64.5	53.1	67.1	71.9
Zhao et al. (2019)	No	88.7	57.7	73.2	65.6	48.9	46.6	51.9	60.8
Ci et al. (2019)	No	60.2	78.9	51.2	50.0	54.8	64.8	43.3	52.7
Liu et al. (2020)	No	60.4	71.1	51.5	50.1	54.5	40.4	43.7	52.4
Xu & Takano (2021)	No	59.7	71.5	51.4	48.6	53.9	40.3	44.1	51.9
Zhao, Wang & Tian (2022)	No	60.2	70.0	51.6	48.7	54.1	39.7	43.1	51.8
Ours	No	45.7	60.8	43.3	41.1	49.6	37.2	41.4	43.4

**Table 7:** Comparison with state-of-the-art fusion-based methods on the Total Capture dataset. “Views” and “IMUs” represent the employed number of cameras and IMUs in these methods, respectively. “Temporal” demotes whether the temporal information from videos is utilized. “–” indicates the results are not reported in the original article.

**Method**	**Views**	**IMUs**	**Temporal**	**SeenSubjects(S1,2,3)**			**UnseenSubjects(S4,5)**			**Mean**
				**W2**	**A3**	**FS3**	**W2**	**A3**	**FS3**	
Trumble et al. (2017)	8	13	No	30.0	49.0	90.6	36.0	109.2	112.1	70.0
Malleson et al. (2017)	8	13	No	–	–	65.3	–	64.0	67.0	(62.0)
Von Marcard et al. (2018)	1	6	Yes	–	–	–	–	–	–	39.6
Gilbert et al. (2019)	8	13	No	19.2	42.3	48.8	24.7	58.8	61.8	42.6
Ours	1	6	No	23.8	36.2	62.4	24.0	48.8	61.7	42.4

#### Comparisons with fusion-based methods

As mentioned above, the work on fusing sparse IMUs and a single-view camera is hardly addressed in the community. To provide a reference of our approach to other methods, the experiment follows the protocol of train and test partitions introduced by Trumble et al. (2017) on the Total Capture dataset. The comparison is regardless of the same number of IMUs or multiple viewpoints and employs the MPJPE in mm as the evaluation metric. The detailed results of these similar works are listed in Table 7. The employed numbers of camera viewpoints and IMUs are also given for clarity. As shown in Table 7, the presented method is superior to the learning-based approach introduced by Trumble et al. (2017), which uses all eight cameras and fuse IMU data with the probabilistic visual hull (PVH). The proposed method also outperforms that of Malleson et al. (2017), which report the MPJPE in 62 mm using eight cameras and all 13 IMUs. The performance of the proposed method is inferior to that of Von Marcard et al. (2018) by 2.8 mm. The main reason is that the method of Von Marcard et al. (2018) is based on the video to model the time information and additional association of 2D and 3D pose. However, the proposed method does not need the extra 2D pose detection compared to Von Marcard et al. (2018), which would make this approach take less time for online applications.

## Conclusion and Future Work

This article proposes a novel learning-based method that combines sparse IMUs and single-frame images to realize 3D human pose and body reconstruction. This proposed method adopts a dual-stream network to extract the IMU and image feature information. The sparse IMU provides rotation information of the terminal joint, which makes up for the significant estimation error of 3D human body reconstruction due to image occlusion. At the same time, the image provides drift-free 3D global position information. Especially, a residual model-attention network is proposed to aggregate dual-modal feature fusion. A final regression network is used to generate 3D pose and shape parameters. By combining the image-based and IMU-based methods, the challenging problem of noisy inertial data and occlusion image is solved in an end-to-end learning manner in practice. Extensive experiments on two public benchmarks demonstrate that the proposed method could effectively fuse sparse IMU data and images and improve pose accuracy. The superiority of the lower error on both human mesh and joint shows that the proposed method can balance the problem of hardware dependence on complexity and precision.

Current applications include games, biomechanical analysis, and human–computer interaction, such as virtual and augmented reality (VR/AR), which impose three challenging limitations on human posture reconstruction, *i.e.,* working in daily environments, minimally invasive instruments for users, and real-time operation. The proposed method relays on single-view images and sparse IMUs that could be easier adapted to the outdoors or in indoor scenes across multiple rooms. In addition, the sparse wearable sensors are less invasive to users, and the single-view based setup can be flexibly deployed through supervision or moving cameras. A limitation of this work is the lack of the estimation of the global translation of human motion. Current work focuses on adopting a standardized SMPL model without fitting the accurate human shape parameters during the initial calibration of the IMU. In future work, we will explore the visual temporal manner including structure from motion to solve the above problems.

## Additional Information and Declarations

### Data Availability

The following information was supplied regarding data availability: The code is available at Gihub and Zenodo: https://github.com/lxxhhh-26/Reconstructing-3D-human-pose-and-shape (DOI: 10.5281/zenodo.7726703). lxxhhh-26. (2023). lxxhhh-26/Reconstructing-3D-human-pose-and-shape: V1.0.1 (V1.0.1). Zenodo. https://doi.org/10.5281/zenodo.7726703
The experiments were conducted using two public datasets: - Human3.6M: http://vision.imar.ro/human3.6m/description.php
- Total Capture: https://cvssp.org/data/totalcapture.
